# The influence of a relict distribution on genetic structure and variation in the Mediterranean tree, *Platanus orientalis*

**DOI:** 10.1093/aobpla/plz002

**Published:** 2019-01-30

**Authors:** Rosita Rinaldi, Donata Cafasso, Sandro Strumia, Antonietta Cristaudo, Federico Sebastiani, Silvia Fineschi

**Affiliations:** 1 Dipartimento di Biologia, Università degli Studi di Napoli Federico II, complesso Universitario Monte Sant’Angelo, via Cinthia, Napoli, Italy; 2 Dipartimento di Scienze e Tecnologie Ambientali, Biologiche e Farmaceutiche, Università degli Studi della Campania L. Vanvitelli, Via Vivaldi, Caserta, Italy; 3 Dipartimento di Scienze Biologiche, Geologiche e Ambientali, Università di Catania, Catania, Italy; 4 Istituto per la Protezione Sostenibile delle Piante, Consiglio Nazionale delle Ricerche, Sesto Fiorentino, I-50019 Firenze, Italy

**Keywords:** Genetic diversity, hybridization, marginal populations, nuclear simple sequence repeats, range fragmentation, relict species, species distribution, Tertiary flora

## Abstract

The distribution of plant species around the Mediterranean basin is a product of the influence of both geographical barriers and of climatic changes experienced during the Tertiary, with the transition from a warm to cool periods. Several species, once largely distributed across the Northern Hemisphere, retracted to refugial areas in southern Europe where they are described as Tertiary relicts. *Platanus orientalis* is a typical representative of Tertiary flora in southwest Eurasia; its distribution spreads from the Caucasus to the Mediterranean, with its western border in Southern Italy and Sicily. We analysed genetic diversity and differentiation in the centre and western part of its distribution range using nuclear microsatellites and compared genetic parameters between core and western populations. We found an overall decrease in genetic diversity estimates (heterozygosity, private allelic richness) from central towards western populations, with those from Southern Italy and Sicily showing the lowest values. The low level of genetic diversity probably results from historic range fragmentation experienced by *P. orientalis* in its westernmost distribution as confirmed by high level genetic isolation of these populations. Ornamental hybrids were genetically distinguished from *P. orientalis* as contained private alleles, indicating that gene flow with natural populations is rare. Population assignment and neighbour-joining (NJ) analysis of populations identified four groups belonging to two main phyletic groups (the Southern Italian-Sicilian-Balkan and Cretan-Bulgarian-Turkish lineages) that seem to have different biogeographic origin and also excluded an artificial origin for southern Italian and Sicilian populations as previously suggested. These results show that quantifying the genetic variation of a Tertiary relict in a geographical context, and the potential effect of hybridization with introduced ornamental hybrids, can provide useful insights on factors influencing population genetic structure. Such information is crucial to predict how such taxa will respond to increasing anthropogenic influence on the environment.

## Teaser

Quantifying the genetic diversity of species is important for understanding their evolutionary history. We investigated the genetic diversity of a Tertiary relict, the tree *Platanus orientalis*, which has an unusual distribution in the Mediterranean with its largest populations in the east, and it is increasingly rare in the west. We found an overall decrease in genetic diversity from eastern to western populations, with populations in southern Italy containing the lowest levels of genetic diversity. As *P. orientalis* requires habitats with high moisture levels, these results provide crucial genetic information which may influence its potential to respond to environmental change.

## Introduction

Geographical barriers play a key role in determining genetic divergence and species distributions (Slatkin 1987). Signatures of past species distribution predating the development of most recent geographic barriers can be detected, even after wide changes in distribution range, through the fossil record, or indirectly from paleoecological and phylogenetic data (Abellán and Ribera 2017). In addition to geographic barriers, climate conditions also determine changes in species ranges and the evolution of locally adapted ecotypes (Araújo and Pearson 2005). In particular, the most recent climatic oscillations in the Quaternary have significantly shaped the present distribution of species in temperate habitats (Petit *et al.* 2005; Médail and Diadema 2009). Consequently, the most recent role of last glacial refugial areas and postglacial migrations has been investigated (Taberlet *et al.* 1998), whereas the effects of previous Quaternary glacial-interglacial cycles and climatic transition from Tertiary are poorly known (Magri *et al.* 2007). These latter events have been particularly important in shaping the distribution range of some plant communities, including several tree species, today confined around the Mediterranean basin but once largely distributed across the Northern Hemisphere (Milne and Abbott 2002). At the end of the Tertiary warm phase (~15 Myr ago) a cooling climate resulted in a retreat of these communities from higher latitude circumboreal distributions southwards to refugial regions characterized by warm and wet climates, where survivors from these communities are now described as Tertiary relicts (Cowling *et al.* 1996). They presently occur in specific areas in the Northern Hemisphere, such as south-eastern and western North America, East Asia and southwest Eurasia (Tiffney 1985; Wen 1999) with a fragmented distribution once connected through land bridges (Milne 2006).

The current distribution of relict Tertiary plants has been constrained by their intrinsic limitation in migrating and colonizing new environments, and today is also threatened by the recent anthropogenic influence on their habitat (Kozlowski *et al.* 2014). In a scenario of global changing conditions, species and populations unable to respond through phenotypic plasticity or genetic adaptation to climate change are at increased risk of extinction (Aitken *et al.* 2008). Indeed, species survival is determined by both migration and adaptive potential to novel conditions within an appropriate time frame. These features ultimately depend on the species’ genetic diversity and range contractions and expansions experienced in the past (Parmesan 2006; Hamilton and Miller 2015). Plant populations confined at the species’ edge are often characterized by smaller size, lower density and reduced connectivity in comparison with core populations as a consequence of suboptimal environmental conditions (Brown *et al.* 1995). Because of this, marginal populations are expected to have lower genetic diversity, higher level of population differentiation and to be more prone to extinction compared with those in the central part of their distribution (Lennon *et al.* 1997; Volis *et al.* 2016). Low genetic diversity may strongly limit the adaptive potential for range expansion/shift in response to environmental changes and, for relict species, increases risk of extinction in a changing environment (Bridle and Vines 2007; Aitken and Bemmels 2015).

*Platanus orientalis*, the oriental plane, is representative of the Tertiary flora in southwest Eurasia, distributed along river courses from the central Mediterranean to the Caucasus and India (Barstow and Rivers 2017). The southern part of the Italian peninsula (hereafter Southern Italy) and Sicily represent the western border with few marginal populations, which are now threatened by human exploitation for agricultural purposes and by habitat destruction (Caruso *et al.* 2012), as occurs in other sites within its range (Barstow and Rivers 2017) as well as by alien pathogens (e.g. the North American fungus *Ceratocystis platani*) (Panconesi 1981). How migration and gene flow have influenced patterns of genetic variation across the present range of this relict tree is unknown. It is also unknown whether gene pools of core and marginal populations are differentiated as a consequence of geographic isolation, or whether its wind pollination strategy is maintaining genetic connectivity among distant populations. *Platanus orientalis* is, among long-lived tree species, one with the largest leaves in the Mediterranean. Consequently, it has been spread by humans across the Mediterranean for ornamental purposes (mostly for shadow provided for its large canopy) during Greek and Roman times (Rosati *et al.* 2015) and Renaissance (Ciaffi *et al.* 2018). Thus, the actual distribution of this tree may be also explained by the strong human contribution to its dispersion. This contribution is predicted to leave a signature on the local level of genetic diversity as artificially introduced populations typically undergo severe genetic bottlenecks (Allendorf and Lundquist 2003). According to this hypothesis, very low/absence of genetic variation in western populations would provide strong evidence for their introduction by humans. Finally, in the last three centuries *Platanus hispanica*, the hybrid between *P. orientalis* and *Platanus occidentalis* (formerly known as *Platanus × acerifolia*), has been widely planted as an ornamental tree providing shade in cities and along main roads, in part due to its resistance to abiotic stresses (Swoczyna *et al.* 2015) and disease (Vigouroux and Olivier 2004; Pilotti *et al.* 2009; Ciaffi *et al.* 2018) as well as to its ability in removing air pollution (Yang *et al.* 2015). Its current widespread occurrence also within the *P. orientalis* range questions whether introgression from introduced hybrid stands may accelerate extinction of native plane tree populations by genetic erosion (Johnson *et al.* 2016).

To our knowledge, the genetic variation and differentiation of *P. orientalis* has not yet been investigated on a large geographical scale. In the present study, by using nuclear microsatellites, we investigated genetic diversity and differentiation in a large and representative portion of *P. orientalis* range, in order to understand whether current fragmented distribution has affected genetic variability and genetic structure in core and western peripheral populations. In particular we aimed to (i) test whether genetic diversity decreases and genetic differentiation increases in marginal populations compared with those in the core of the species range, (ii) determine genetic diversity and connectivity of core and marginal populations, (iii) identify patterns of phyletic relationships among core and marginal populations and (iv) estimate potential hybridization with introduced ornamental hybrids.

## Materials and Methods

### Study species

*Platanus* is the only living genus of the Platanaceae. Seven living *Platanus* species are distributed with the classic disjunct pattern of Tertiary relict taxa around the Northern Hemisphere (Feng *et al.* 2005) and *P. orientalis* is the only extant native species in Europe (Tutin and Edmondson 1993).

*Platanus* fossil records are abundant and demonstrate that Platanaceae were once widespread in Europe since the early Cretaceous. During the Tertiary, the family consisted of several taxa that are now extinct, including forms with compound leaves, which are unlike extant species (Kvaček and Manchester 2004). Tertiary macrofossils show that now extinct *Platanus* species occurred in northern, central and southern Europe (Tschan *et al.* 2008; Velitzelos *et al.* 2014). At the end of the Tertiary (Pliocene) and the beginning of the Quaternary, likely as a consequence of Pleistocene glaciations, *Platanus* pollen and macrofossils become very sporadic in France (Argant 2004), Iberian peninsula (Postigo-Mijarra *et al.* 2010), Italy (Bertini *et al.* 2010) and Greece (Velitzelos *et al.* 2014). We examined *P. orientalis* populations from both its range core and from isolated, marginal populations that are representative of the westernmost edge of the species distribution in Southern Italy and Sicily (Barstow and Rivers 2017) (Table 1). In these latter regions we sampled wild sites reported in historic records (Beguinot 1925; Caruso *et al.* 2012 and reference therein). All *P. orientalis* sampled individuals were identified based on morphological characters according to (Tutin and Edmondson 1993).

**Table 1.** Sampled populations of *Platanus orientalis*, including region of origin, coordinates and sample size (*N*).

Populations/code	Region	Lat	Long	*N*
Alento/ALE	S-Italy	40.277	15.126	112
Velia/VEL	S-Italy	40.131	15.134	15
Uria/CAL	S-Italy	38.912	16.725	20
Cosenza/COS	S-Italy	39.175	16.151	5
Alcantara/ALC	Sicily	37.895	15.079	7
Anapo/ANA	Sicily	37.094	15.170	37
Cataldo/CAT	Sicily	37.534	15.113	20
Manghisi/MAN	Sicily	36.590	15.014	12
Augeniki/AUG	Crete	35.111	25.211	15
Gortis/GOR	Crete	35.256	25.122	13
Nestos/NES	Greece	41.125	24.400	10
Acherontas/ACH	Greece	39.329	20.604	44
Vjose/VJA	Greece	40.031	20.636	12
Aoos/AOO	Greece	40.030	20.700	10
Osum/OSU	Albania	40.803	19.867	14
Drino/DRI	Albania	40.000	20.257	31
Vjose/VJB	Albania	40.152	20.491	2
Kresna/KRE	Bulgaria	41.431	23.095	10
Topolovo/TOP	Bulgaria	41.540	25.003	10
Mikrevo/MIK	Bulgaria	41.372	23.114	11
Dardanelles/DAR	Turkey	40.623	26.248	11
Beskonak/BES	Turkey	40.373	32.412	8
Total				429

### DNA extraction and simple sequence repeat analysis

Genomic DNA was extracted from 50 mg of silica gel-dried leaf tissue using the Qiagen DNeasy™ Plant Mini Kit (Valencia, CA, USA). Seven loci previously developed for *P. occidentalis* (Lang 2010) and two specifically developed for *P. orientalis* for a total of nine nuclear microsatellites were employed (Table 2). The PCR reaction mixture consisted of 10 μL total volume containing 20 ng of genomic DNA. Cycling parameters were the following: 3 min at 94 °C, 30 cycles of 30 s at 94 °C, 60 s ranging from 50 to 62 °C (depending on the primer annealing temperature; Table 2), 1 min at 72 °C and a final step of 7 min at 72 °C. Amplified microsatellite products were genotyped using ABI 3130 sequencer and sized in accordance with LIZ500 standard by using GENEMAPPER software v.3.7 (Thermo Fisher Scientific-Applera, Waltham, Massachusetts, USA).

**Table 2.** Characteristics of the nine polymorphic microsatellites in *Platanus*: *N*_a_, total number of alleles per locus; *T*_a_, annealing temperature; *Dye linked with forward primer in each case.

Locus	Primer sequence 5′-3′	Repeat motif	Size range (bp)	*N*_a_	Dye*	*T*_a_ (°C)	PCR cycles
plms29^[1]^	F: GCCCATTAGATGGGTTGAAA R: AGCGAATCCATGTGCCTAAT	(TC)_9_	208–224	5	Hex	58	35
plms68^[1]^	F: TGAATCCCAAAAGGCAAAAA R: AAACACCCAATCCGGTCTAC	(GT)_8_(AT)_2_(GT)_5_	170–184	3	Fam	50	35
plms71^[1]^	F: ACGGGTGAGCTCCCTACTTT R: GACATCCTCCACCAAACACC	(TG)_10_	135–137	2	Hex	60	35
plms109^[1]^	F: TGATGACAAATACTCAGGGAAA R: CGATAGCCAAAAGCGAAAGA	(CA)_18_	121–145	5	Atto 550	60	35
plms113^[1]^	F: GGCAAGCCAGGATTTAGTTG R: CGGGATAAGAGTTTGTTGAGTTG	(CT)_11_(CA)_16_	202–228	11	Atto 550	62	30
plms130^[1]^	F: TACCACACCAACGTCCTTCC R: ACCCTCTCAAATATGCCAATTA	(CA)_7_	208–214	4	Atto 565	58	35
plms176^[1]^	F: AACAGCAAAACAGCCCACTC R: AAACCAGCCAATCCAATTCC	(CA)_9_	269–275	6	Atto 565	60	30
11FAM^[2]^	F: TCGGTGGTCGAATTCATCCC R: GAAAGCATGCCGATGTGGG	(TC)_18_	202–236	16	FAM	53	35
PI2A^[3]^	F: GAGGGAAGGATTGCCCAGTTG R: CTATCAACTTCTAGATCCCTAG	(CT)_22_	332–354	10	NED	53	35

^[1]^ Lang (2010)—Microsatellite development in *Platanus* for documenting gene flow among species. Thesis in Biological Sciences; California State University.

^[2]^Microsatellites Library (unpublished).

^[3]^From GenBank accession number: HM196359.

### Genetic diversity estimates

A total of 429 specimens from 22 populations were genotyped. The genetic diversity of each population was estimated by using the number of alleles (*A*), allelic richness (*Rs*), private allelic richness (*PA*), expected (*H*_E_) and observed (*H*_O_) heterozygosity, and the inbreeding coefficient *F*_IS_, calculated using the programs msa v. 4.05 (Dieringer and Schloetterer 2003) and hp-rare v. 1.0 (Kalinowski 2005). We tested for a correlation between heterozygosity (*H*_E_) and longitudinal distribution using the Pearson correlation coefficient. Departures from Hardy–Weinberg equilibrium (HWE) due to within-population inbreeding were estimated using exact tests in Genepop v. 4.0 (Raymond and Rousset 1995). The microsatellite data set was tested for the presence of null alleles using the program ML-NullFreq (Kalinowski and Taper 2006). In order to minimize the effects of clonal propagation on estimates of genetic diversity and population differentiation repeated multilocus genotypes (MLGs) were identified using Genodive 2.0b7 (Meirmans and Van Tienderen 2004). Clone correction (removal of clones) reduced the number of compared MLGs from 429 to 373 (in particular, the Alento population was reduced from 112 to 77 samples). All subsequent analyses were performed on the clone-corrected data set (Table 3).

**Table 3.** Sample size (*N*) after clone correction identified using Genodive 2.0b7; number of alleles (*A*); allelic richness calculated with the rarefaction method (*Rs*); private alleles richness (*PA*); unbiased expected heterozygosity (*H*_E_); observed heterozygosity (*H*_O_); inbreeding coefficient (*F*_IS_). Significant values for *F*_IS_ (*P* < 0.05) are in bold, after sequential Bonferroni correction*.

Populations/code	*N*	*A*	*Rs*	*PA*	*H*_E_	*H*_O_	*F*_IS_
Alento/ALE	77	1.850	1.764	0.068	0.370	0.207	**0.442**
Velia/VEL	9	1.713	1.763	0.088	0.402	0.346	0.147
Uria/CAL	15	1.573	1.537	0.002	0.270	0.252	**0.069**
Cosenza/COS	5	1.426	1.477	0.000	0.267	0.267	0.000
Alcantara/ALC	7	1.543	1.589	0.016	0.292	0.286	0.023
Anapo/ANA	30	1.619	1.574	0.021	0.286	0.270	**0.057**
Cataldo/CAT	20	1.569	1.588	0.007	0.301	0.256	**0.154**
Manghisi/MAN	11	1.652	1.584	0.001	0.296	0.273	**0.082**
Augeniki/AUG	15	2.675	2.292	0.097	0.607	0.550	**0.098**
Gortis/GOR	13	2.740	2.224	0.120	0.557	0.564	−0.013
Nestos/NES	10	1.921	1.930	0.103	0.448	0.289	**0.368**
Acherontas/ACH	42	1.911	1.740	0.027	0.348	0.214	**0.387**
Vjose/VJA	12	2.205	1.892	0.127	0.411	0.269	**0.358**
Aoos/AOO	10	2.113	1.941	0.141	0.456	0.284	**0.391**
Osum/OSU	14	2.120	1.772	0.037	0.362	0.302	**0.173**
Drino/DRI	31	2.403	1.919	0.066	0.427	0.252	**0.413**
Vjose/VJB	2	1.563	1.778	0.027	0.426	0.389	0.167
Kresna/KRE	10	2.225	2.012	0.018	0.495	0.467	0.060
Topolovo/TOP	10	2.206	1.971	0.048	0.468	0.423	0.100
Mikrevo/MIK	11	2.282	2.036	0.071	0.475	0.414	**0.133**
Dardanelles/DAR	11	2.213	2.037	0.039	0.494	0.545	−0.110
Beskonak/BES	8	2.219	1.985	0.219	0.472	0.452	0.045
**Total**	**373**	**1.988**	**1.837**	**0.061**	**0.406**	**0.344**	**0.159**

*As the sample size of most of the populations was <15 individuals, *F*_IS_ should be treated with caution.

### Population structure and demography

Population genetic structure was characterized by estimating pairwise *F*_ST_ among populations with Genodive 2.0b7 (Meirmans and Van Tienderen 2004). The significance of *F*-statistic estimates was assessed using 10 000 permutations. To verify that obtained *F*_ST_ estimates are not an artefact of high intra-population diversity, we also compared differentiation using the population differentiation estimate *D*_ST_ (Jost 2008). Partitioning of genetic diversity was examined at different hierarchical levels using the analysis of molecular variance (AMOVA) implemented in the software arlequin v. 3.5 (Excoffier and Lischer 2010). Analysis of molecular variance was also used to explore the genetic variation partitioning when populations were grouped according to the region of origin.

Population structure was evaluated using a Bayesian clustering algorithm of individuals implemented in the software STRUCTURE v. 2.3.4 (Pritchard *et al.* 2000). As the Alento and Velia populations were found characterized by a high number of clonal individuals and were supposed to have a partial artificial (clonal) origin, we run STRUCTURE simulations by both including and excluding these clonal individuals from the data set.

Ten runs were performed for each value of *K*, from *K* = 2 to 10, with burn-in lengths of 100 000 and 100 000 iterations. STRUCTURE HARVESTER (Earl and von Holdt 2012) was employed to calculate the probability of the data for each *K* and to calculate *DK* according to the method described by Evanno *et al.* (2005). STRUCTURE PLOT program (Ramasamy *et al.* 2014) was utilized to visualize the STRUCTURE output.

Pairwise genetic distances between populations were estimated using the program Populations version 1.2.31 (Langella 2002). A neighbour-joining (NJ) tree constructed from the *D*_*A*_ (Nei *et al.* 1983) matrix was used to represent the relationships among groups by bootstrapping (1000 replicates) distance values over loci.

The intensity of gene flow is crucial to explain current patterns of genetic structure and is of particular interest for small and/or isolated populations. Theta (4*N*_e_µ for biparental inherited loci, with *N*_e_ = effective population size and µ = mutation rate) and the number of immigrants per generation (θ*M*, with *M* = the mutation-scaled effective immigration rate) were estimated under a coalescent framework using the program Migrate-n 3.6.4 (Beerli and Felsenstein 2001; Beerli 2006). In order to estimate *N*_e_ we used the mutation rate of 0.00077 detected in maize microsatellites (Vigouroux *et al.* 2002). Starting values were calculated using *F*_ST_, and we used model averaging to estimate migration rates and θ values. Migrate-n analyses were conducted using a static heating strategy with four short chains (with temperature values of 1.0, 1.5, 3.0 and 1.0 × 10^6^) and a single long chain with 50 000 recorded steps, an increment of 50 and 20 000 steps discarded as burn-in. The number of concurrent chains (replicates) was 10. Stationarity of the Markov chain was assessed by examining the effective sample size for each parameter.

### Estimation of genetic introgression and current gene flow with introduced *P. hispanica*

Based on allelic difference between *P. orientalis* and *P. hispanica* at microsatellite loci employed both in Lang (2010) and in the present study, genotype profiles of all examined *P. orientalis* individuals were scored for the presence of putative *P. hispanica* alleles.

Based on this first screening, the amount of ongoing pollen flow between a natural stand of *P. orientalis* and surrounding *P. hispanica* trees was estimated in the Alento population. For this, ~1400 pooled seeds from 10 genotyped *P. orientalis* mother plants from the Alento population were germinated on sterilized sand. After 2 months, 239 plantlets were sampled (first two leaves) and genotyped as described above to detect occurrence of putative *P. hispanica* alleles in the seed progeny. Additionally, we also genotyped a set of Southern Italian *P. hispanica* plants, both of nursery and of street origin. In particular, two specimens were collected at the closest proximity (1 km) to the natural *P. orientalis* Alento population.

## Results

### Genetic diversity estimates

**Figure 1.**
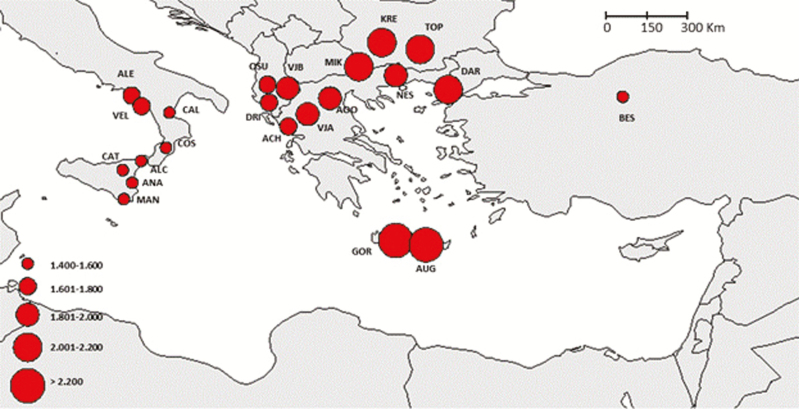
Spatial distribution of genetic diversity (allelic richness) standardized to the smallest sample size (population).

**Figure 2.**
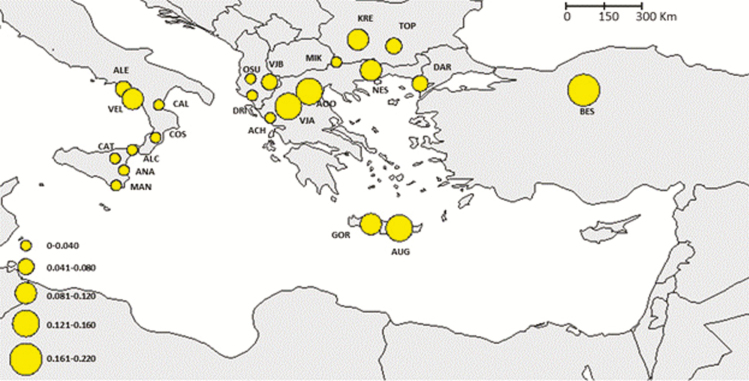
Spatial distribution of genetic diversity (private allelic richness) standardized to the smallest sample size (population).

**Figure 3.**
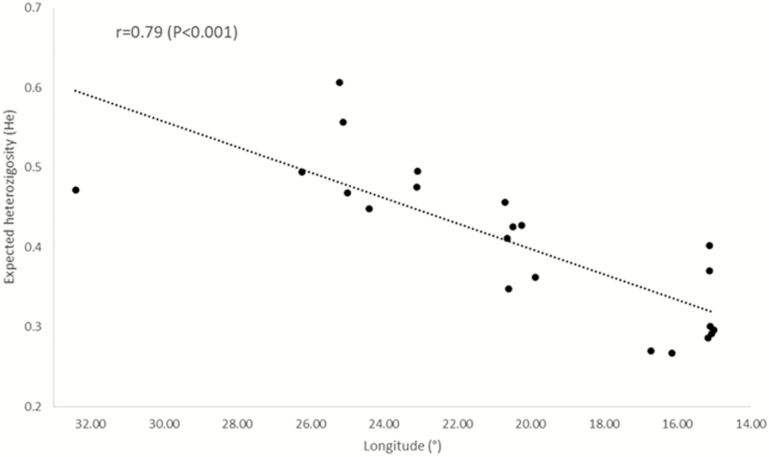
Pearson correlation (and linear trend) between longitude (°) and heterozigosity (*H*_E_).

Clonal individuals were detected in some populations, particularly in the Alento population. Here, several individuals share an identical microsatellite profile suggesting a clonal propagation from local wild stock (Table 3). In general, genetic diversity estimates showed decreasing values from eastern towards western populations, the Southern Italian and Sicilian ones showing the lowest values. Alleles richness and private allelic richness followed the decreasing trend from eastern to western populations, with values ranging between 1.477 to 2.292 and 0.000 and 0.219, respectively (Table 3; Figs 1 and 2). The population Beskonak in Turkey showed the highest number of private alleles, while the lowest number of private alleles was observed in the Italian population Cosenza, which was also characterized by the lowest allelic richness. The Cretan populations displayed the highest allelic richness as well the highest expected and observed heterozygosity, which ranged from 0.267 (Cosenza, Italy) to 0.607 (Augeniki, Crete) and between 0.207 (Alento, Italy) and 0.564 (Gortis, Crete), respectively (Table 3). Indeed, the progressively decline in expected heterozygosity was significantly correlated an eastern to western longitudinal gradient (*R* = 0.79; *P* ≤ 0.0001; Fig. 3).

Inbreeding depression was high and significant for most populations, ranging between 0.000 (Cosenza) and 0.442 (Alento). In general, the lowest values of inbreeding depression were detected at the western (Southern Italy) and eastern (Turkey, Bulgaria) parts of the distribution of *P. orientalis* (Table 3). The presence of null alleles was ruled out by ML-NullFreq tests.

### Population structure, gene exchange and historical size reduction

Significant levels of nuclear genetic differentiation among populations (*P* < 0.001) were found for *F*_ST_ (0.209) and *D*_ST_ (0.183). Most of the pairwise *F*_ST_ values were significant, and similar values were obtained by pairwise *D*_ST_ analysis (Table 4). Higher *F*_ST_ values were found between marginal populations from Southern Italy and Sicily while lower *F*_ST_ values were found between populations from Crete (Augeniki and Gortis), Balkans (Vjose, Aoos, Osum, Drino) and Bulgaria (Mikrevo, Kresna, Topolovo). Analysis of molecular variance revealed that a significant proportion of the genetic diversity was found within populations (79.0 %, *P* < 0.0001), while 21.0 % genetic diversity was found between populations. The AMOVA performed after grouping populations according to the regions of origin gave similar results, with 12.0 % of the variation distributed between groups, 25.9 % between populations within groups and 62.1 % within populations (*P* < 0.0001 for all hierarchical levels).

**Table 4.** Pairwise comparisons of *F*_ST_ (below diagonal) and *D*_ST_ (above diagonal) between populations of *Platanus orientalis* based on nine simple sequence repeats. Values given in bold are significant at *P* < 0.001.

	ALE	VEL	CAL	COS	ALC	ANA	CAT	MAN	AUG	GOR	NES	ACH	VJA	AOO	OSU	DRI	VJB	KRE	TOP	MIK	DAR	BES
ALE	0.000	0.075	0.109	0.172	0.176	0.170	0.167	0.230	0.189	0.232	0.246	0.160	0.167	0.193	0.182	0.179	0.136	0.236	0.263	0.240	0.286	0.385
VEL	**0.107**	0.000	0.175	0.187	0.261	0.201	0.216	0.241	0.212	0.292	0.229	0.225	0.179	0.192	0.227	0.221	0.253	0.257	0.239	0.272	0.216	0.313
CAL	**0.169**	**0.268**	0.000	0.234	0.258	0.175	0.222	0.219	0.221	0.326	0.260	0.092	0.126	0.124	0.112	0.124	0.060	0.203	0.329	0.283	0.300	0.388
COS	**0.231**	**0.254**	**0.387**	0.000	0.279	0.218	0.156	0.175	0.239	0.253	0.406	0.293	0.223	0.252	0.263	0.287	0.316	0.288	0.354	0.370	0.297	0.347
ALC	**0.236**	**0.321**	**0.400**	0.416	0.000	0.103	0.048	0.117	0.154	0.156	0.150	0.176	0.168	0.204	0.170	0.174	0.177	0.110	0.177	0.116	0.241	0.333
ANA	**0.245**	**0.299**	**0.310**	**0.356**	**0.203**	0.000	0.086	0.051	0.195	0.213	0.238	0.194	0.101	0.165	0.156	0.183	0.196	0.154	0.166	0.196	0.216	0.260
CAT	**0.234**	**0.297**	**0.354**	**0.270**	0.100	**0.171**	0.000	0.027	0.123	0.136	0.234	0.142	0.090	0.130	0.102	0.143	0.154	0.129	0.211	0.202	0.225	0.288
MAN	**0.294**	**0.312**	**0.357**	**0.302**	**0.217**	**0.111**	0.060	0.000	0.163	0.200	0.290	0.152	0.042	0.092	0.079	0.116	0.141	0.122	0.200	0.236	0.198	0.252
AUG	**0.192**	**0.162**	**0.221**	**0.193**	**0.136**	**0.219**	**0.136**	**0.156**	0.000	0.012	0.155	0.177	0.128	0.114	0.153	0.144	0.056	0.075	0.121	0.150	0.112	0.205
GOR	**0.238**	**0.234**	**0.322**	**0.230**	**0.158**	**0.254**	**0.163**	**0.207**	0.008	0.000	0.217	0.249	0.197	0.205	0.240	0.222	0.151	0.108	0.134	0.141	0.156	0.206
NES	**0.276**	**0.231**	**0.327**	**0.388**	0.190	**0.319**	**0.295**	**0.327**	**0.116**	**0.173**	0.000	0.217	0.268	0.210	0.242	0.237	0.185	0.132	0.213	0.132	0.259	0.380
ACH	**0.219**	**0.280**	**0.159**	**0.365**	**0.253**	**0.291**	**0.222**	**0.230**	**0.183**	**0.256**	**0.262**	0.000	0.054	0.033	0.018	0.023	−0.090	0.109	0.304	0.183	0.300	0.355
VJA	**0.212**	**0.203**	**0.199**	**0.272**	**0.221**	**0.172**	**0.145**	0.069	**0.106**	**0.171**	**0.257**	**0.085**	0.000	−0.010	0.001	0.014	−0.047	0.101	0.204	0.203	0.187	0.257
AOO	**0.230**	**0.199**	**0.187**	**0.276**	**0.239**	**0.245**	**0.189**	**0.133**	**0.087**	**0.163**	**0.197**	0.054	−0.012	0.000	−0.007	0.008	−0.082	0.073	0.269	0.208	0.211	0.293
OSU	**0.236**	**0.269**	**0.194**	**0.341**	**0.248**	**0.254**	**0.172**	**0.136**	**0.138**	**0.221**	**0.263**	0.031	0.003	−0.007	0.000	0.010	−0.053	0.107	0.302	0.223	0.263	0.325
DRI	**0.217**	**0.230**	**0.173**	**0.303**	**0.207**	**0.246**	**0.192**	**0.153**	**0.125**	**0.193**	**0.233**	0.035	0.018	0.011	0.013	0.000	−0.102	0.066	0.233	0.164	0.194	0.296
VJB	0.176	0.228	0.198	0.466	0.326	0.371	0.235	0.321	0.050	0.121	0.211	−0.117	−0.003	−0.085	−0.019	−0.119	0.000	0.026	0.230	0.069	0.238	0.268
KRE	**0.257**	**0.237**	**0.260**	**0.291**	0.137	**0.221**	**0.177**	**0.159**	0.055	**0.088**	0.127	**0.144**	**0.108**	0.073	**0.128**	0.072	0.029	0.000	0.133	0.064	0.113	0.234
TOP	**0.285**	**0.235**	**0.375**	**0.352**	**0.214**	**0.242**	**0.269**	**0.246**	0.090	**0.111**	**0.198**	**0.326**	**0.204**	**0.234**	**0.302**	**0.224**	0.276	**0.124**	0.000	0.081	0.067	0.174
MIK	**0.265**	**0.254**	**0.332**	**0.353**	**0.146**	**0.268**	**0.255**	**0.271**	**0.109**	**0.115**	**0.131**	**0.223**	**0.201**	**0.189**	**0.238**	**0.168**	0.141	0.063	0.083	0.000	0.170	0.203
DAR	**0.295**	**0.209**	**0.339**	**0.300**	**0.259**	**0.281**	**0.269**	**0.233**	**0.081**	**0.123**	**0.223**	**0.313**	**0.184**	**0.187**	**0.264**	**0.188**	0.228	**0.104**	0.068	**0.153**	0.000	0.110
BES	**0.368**	**0.288**	**0.420**	**0.353**	**0.345**	**0.336**	**0.338**	**0.296**	**0.141**	**0.159**	**0.304**	**0.361**	**0.244**	**0.248**	**0.319**	**0.267**	0.308	**0.199**	**0.163**	**0.183**	**0.106**	0.000

**Figure 4.**
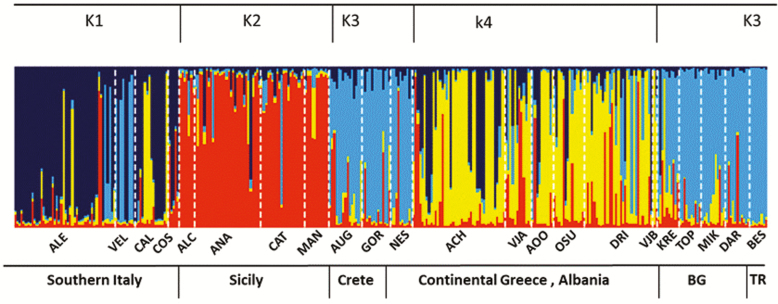
Distruct plot for *Platanus orientalis* populations. Each cluster (*K* = 4) is represented by a different colour. Dotted lines separate populations (population codes below the figure). Populations are grouped into six geographical regions of origins.

**Figure 5.**
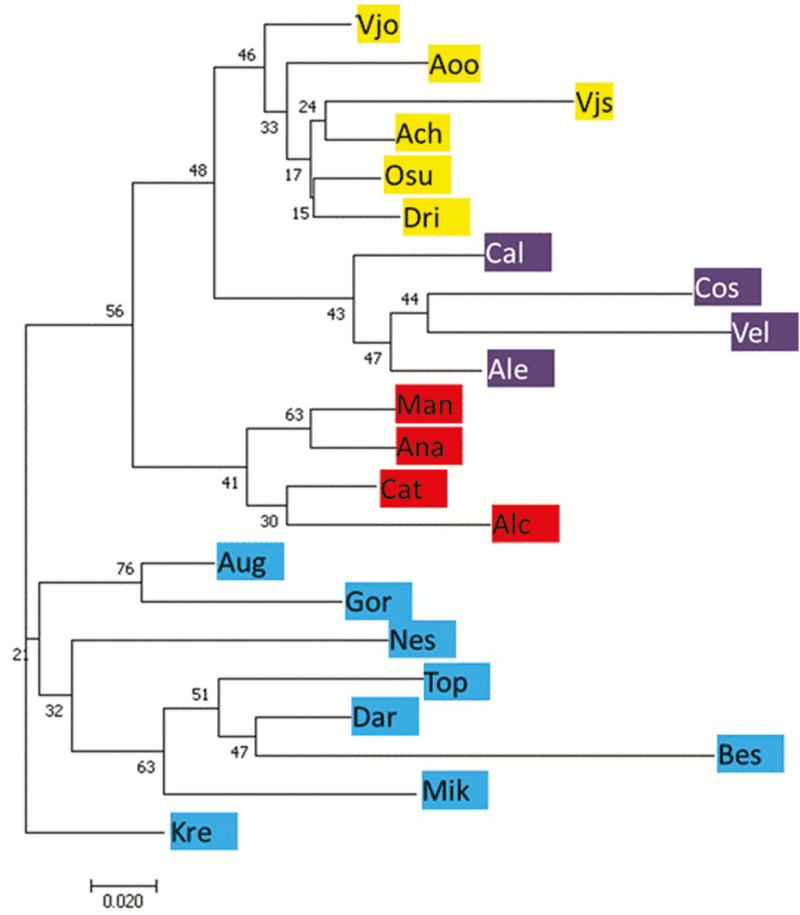
Neighbour-joining (NJ) tree based on Nei genetic distance (*D*_*A*_) among populations for nine microsatellite loci assayed for 373 individuals. Bootstrapping (1000 replicates) distance values over branches. Population coloured labels according to clusters as in Fig. 4. See Table 1 for population codes.

Simulations performed in STRUCTURE consistently identified *K* = 4 clusters after removing the clonal individuals of Alento (*K* = 5 clusters, when included; **see****Supporting Information—Fig. S1**). However, few admixed individuals are present in all populations. The frequencies of each genetic cluster showed marked differences between regions. Two clusters corresponded to the western part of the distribution range of *P. orientalis*, one to Southern Italy and one to Sicily; another cluster included continental Greece and Albania (i.e. Balkans); the fourth cluster included the central range of the species, i.e. Bulgaria, Turkey and Crete (Fig. 4). Some admixture was detected among distant geographic locations, such as Southern Italy and continental Greece and Crete **[see****Supporting Information—Fig. S2****]**. Neighbour-joining tree (Fig. 5) identified four main clades (even if with low bootstrap support) corresponding to the four clusters previously detected with STRUCTURE.

**Figure 6.**
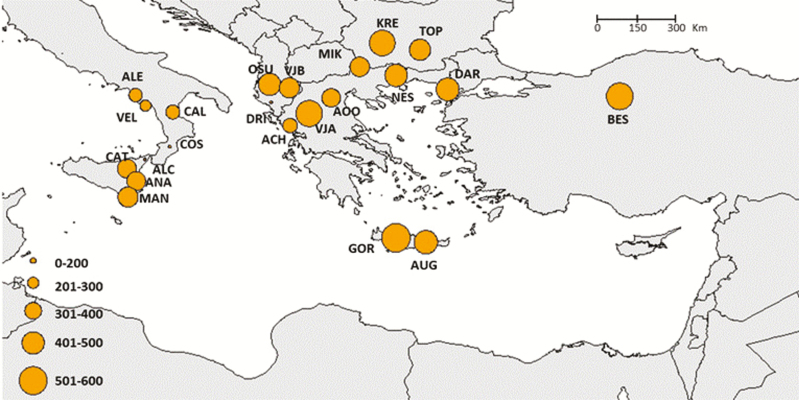
Spatial distribution of effective population size (*N*_e_).

**Figure 7.**
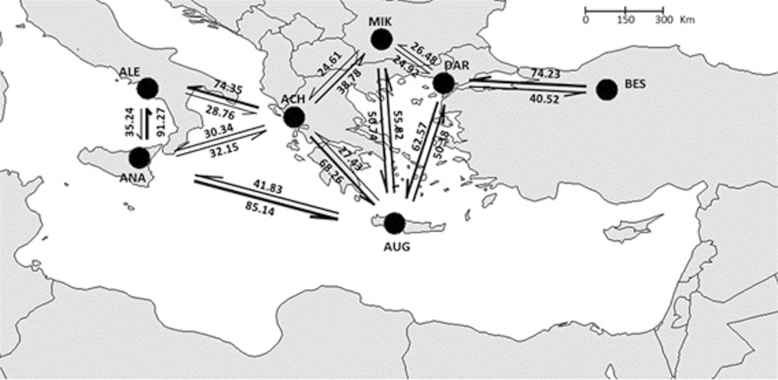
The effective number of immigrants per generation (below and above arrows) calculated for adjacent populations with higher sample sizes. Arrows indicate the direction of gene flow and thickness of lines are according to immigrant number. See Table 1 for population codes.

The effective population sizes ranged from 198 to 512, and most values fell between the interval of 300 and 500 **[see****Supporting Information—Table S1****]**. As observed for the genetic diversity estimates, slightly smaller *N*_e_ values were observed in the western populations (Fig. 6). Results of Migration analyses were obtained for all population pairs (the complete analysis is reported in **Supporting Information—Table S2**), but due to the high amount of comparisons, only results for populations with higher sample sizes and occurring in different regions are shown (Fig. 7). The analysis of migrants per generation displayed values between 20 and 100 for almost all population pairs. Results were asymmetric for most population pairs, but did not identify patterns of preferential gene exchange directions. Island populations generally did not behave as genetic sinks. For example, the intensity of gene exchange was high when Anapo (Sicily) acted as donor to Alento (peninsular Southern Italy). On the other hand, gene exchange was high from Balkan and Sicilian populations towards the Cretan Augeniki population.

### Genetic introgression and current gene flow with introduced *P. hispanica*

In eight individuals (four from ALE, two from DRINO one from ACHE and MIK, respectively) we detected at least one private allele of putative *P. hispanica* origin **[see****Supporting Information—Table S3****]**.

As already reported in Lang (2010), our genotyped *P. hispanica* plants harbour exclusive alleles respect to *P. orientalis* individuals sampled in the present survey **[see****Supporting Information—Table S4****]**. Genotyping of seed progeny in the Alento population revealed that only two out of 239 genotyped plantlets (i.e. 0.8 %) contain private alleles also detected in the neighbouring *P. hispanica* ornamental trees. This indicates there is putative gene exchange via pollen flow between ornamental and natural stands.

## Discussion

We evaluated neutral genetic variation and genetic structure of *P. orientalis* populations along an east-west distributional gradient. Overall, we found a progressive decrease in genetic diversity and connectivity from the core (central) to the marginal (western) populations.

For wind-pollinated tree species, such as *P. orientalis*, gene flow is expected to homogenize the neutral genetic variation among populations, even at a very large geographical scale (Duminil *et al.* 2007). However, the disjunct distribution and the historical and recent range fragmentation experienced by relict species may impact on amount of gene flow between core and marginal populations and on the local distribution of genetic variation (Wójkiewicz *et al.* 2016).

The patterns of genetic diversity we detected in *P. orientalis* populations differ from temperate wind-pollinated tree species, which generally display high genetic diversity, low level of inbreeding and low genetic differentiation among populations (Duminil *et al.* 2007). Indeed, the observed heterozygosity (*H*_o_) was significantly lower than the expected heterozygosity (*H*_E_) in several populations indicating deviation from HWE and significant inbreeding (Table 3). Over the range of *P. orientalis*, the central populations have, on average, higher genetic variability than western marginal ones. In particular, we observed an overall decrease of genetic diversity (heterozygosity, private alleles richness and allelic richness) from core towards marginal populations, with those from Southern Italy and Sicily, at the species western edge, showing the lowest values (Table 3; Figs 1–3 and 6). This pattern is consistent with the central-marginal hypothesis, which is related to the ‘abundant-centre’ hypothesis (Sagarin and Gaines 2002; Sagarin *et al.* 2006). According to this hypothesis, genetic diversity is expected to be lower and genetic differentiation is expected to be higher in marginal populations compared with those in the core of the species range (Duffy *et al.* 2009). This hypothesis was demonstrated in the majority of experimental studies reviewed by Eckert *et al.* (2008); however, some relict species did not display the expected results (e.g. *Liriodendron chinense*; Yang *et al.* 2016). The low level of genetic diversity can result from range fragmentation experienced by *P. orientalis* in its westernmost distribution. When populations become thinned and isolated, the number of pollen donors, thus pollen availability, decreases, leading to reduced reproduction rate and elevated levels of inbreeding (Mimura and Aitken 2007). This pattern is not necessarily determined by geographic distance among populations; indeed, the geographically isolated populations of Crete are characterized by high level of genetic diversity (Fig. 1; Table 3).

Compared to other tree species characterized by the same life history traits (Duminil *et al.* 2007), *P. orientalis* populations are highly genetically differentiated (average *F*_ST_ = 0.209), suggesting that fragmentation represents a strong barrier to gene flow between stands. In particular, pairwise genetic differentiation showed the highest values between the western marginal populations than between the Balkan ones (core population). *F*_ST_ values between marginal populations of Southern Italy and Sicily were even higher than those found between them and the Balkan populations (Table 4). Reduced genetic diversity of *P. orientalis* populations from Southern Italy and Sicily contrasts with those found in other widespread Mediterranean tree species (Médail and Diadema 2009 and reference therein) whose distributions are strongly influenced by climate, particularly temperature (Kovar-Eder and Kvacek 2007). Overall, the historical climate conditions play a crucial role in explaining both current distribution and population genetic diversity of many species. Since the last glacial maximum many species that occurred in the Mediterranean have shifted northwards, and indeed many now only occur in low frequency in the Mediterranean such as *Abies alba* (Linares 2011). However, *P. orientalis* is a typical species found in alluvial plain forests, hence its distribution is more strongly influenced by humidity and water availability compared with other species that shifted north after the previous glacial period. Hence, *P. orientalis* is currently restricted to a specific habitat type (low-altitude valley bottom) which has represented or a glacial temperate refugia in southern Europe for most European tree species (Svenning 2003) or a barrier to migration for other tree species (Magri *et al.* 2006).

Although the Mediterranean region is characterized by high number of endemics, there are only few examples of Tertiary relic tree species with comparable distribution to *P. orientalis*. Among those investigated, *Cupressus sempervirens* has reduced heterozygosity and allelic richness moving from Greece and Turkey towards Italy. These findings suggest that Italian populations experience both severe bottlenecks, which results in reduced genetic diversity, allelic richness and greater genetic differentiation, and recent colonization or introduction from eastern populations (Bagnoli *et al.* 2009).

STRUCTURE simulations identified four groups and indicated partial admixture, thus incomplete differentiation in *P. orientalis*. The identified groups correspond to four geographic regions: Southern Italy, Sicily, Balkans (continental Greece and Albania) and an eastern one that groups together Crete, Bulgaria and Turkey (Fig. 4; **see****Supporting Information—Fig. S2**). The relationships among populations (Fig. 5) were similar to the STRUCTURE results and identified two main phyletic lineages that probably have different biogeographic origins. One lineage includes the southern Italian, Sicily and Balkan regions, which might confirm the occurrence of a continuous distribution through the Adriatic bridge that connected Southern Italy with Balkans (Fineschi *et al.* 2002). The other lineage includes Cretan, Bulgarian and Turkish populations. *Platanus orientalis* at the western edge (Southern Italy and Sicily) displayed higher genetic differentiation and reduced genetic diversity, in terms of allelic richness (*N*_e_), private allele richness, heterozygosity (*H*_O_, *H*_E_) and higher *F*_ST_, than the southern marginal populations of Crete (Table 3; Figs 1 and 2). This result might indicate Crete as a refugial area for *P. orientalis*, where larger population size and higher genetic diversity than at the western edge were probable maintained thanks to a less strong anthropogenic pressure as experienced by river basins of Southern Italy and Sicily with consequent reduction of water level (Surian and Rinaldi 2003).

Reduced diversity at the western margins may be associated with decreased potential to adapt to changing environments. Marginal stands are often characterized by very low natural recruitment. The absence of juveniles might be due to unfavourable ecological factors limiting or inhibiting seedling development (e.g. increasing aridity), or by genetic barriers that hinder pollination and/or fertilization. Kramer *et al.* (2008) and Lowe *et al.* (2005) emphasized the role of ecological and environmental factors in reducing seed set and progeny fitness in fragmented environments. Moreover, Lowe *et al.* (2015) pointed out that understanding fitness consequences of fragmentation requires focusing attention on progeny and on its relative success to assure natural regeneration, rather than on adult populations as commonly done. As also found in other tertiary relict species (Hampe and Arroyo 2002), *Platanus* populations in the western range has almost no regeneration and no/very few juvenile plants were detected during our sampling (authors’ pers. obs.). Even if we have no estimation of potential reproductive success of *P. orientalis* populations in the western range, the absence of natural regeneration prevents the dispersal and colonization potential of these marginal populations. Asymmetrical gene flow from the core to the marginal populations may increase frequency of unfavourable genes in peripheral populations, thus reducing their potential range expansion and local adaptation (Bridle and Vines 2007). However, we did not observe asymmetrical historical gene flow from core *P. orientalis* populations suggesting that the reduced reproductive potential of edge populations is not due to a predominant influx of foreign individuals/alleles that are not adapted to local environmental conditions. Hence, performing reciprocal crosses between central and marginal populations and comparing the performance of seed progeny in terms of germination and seedlings survival could demonstrate the local adaptive limits of this species.

The occurrence of *P. orientalis* in proximity of Greek/Roman archaeological sites, the disappearance of *Platanus* pollen from the Holocene records and its reappearing since the Roman era raises the question whether *P. orientalis* populations from Southern Italy and Sicily have to be considered remnant of ancient introductions (Rosati *et al.* 2015). However, with the notable exception of a part of the Alento population, which has clear signature of clonal propagation from local genetic resources, current levels of genetic diversity and effective population sizes of Southern Italian and Sicilian populations do not appear to be the result of human-mediated introduction. Even if there was a reduction of genetic diversity along a core-peripheral gradient, the genetic variation found in the marginal populations of Southern Italy and Sicily (in terms of private allelic richness and effective population size) is too large to be explained by the artificial introduction of few founder individuals. Indeed, genetic diversity of introduced populations is expected to decline with increasing distance from the source populations because of successive founder events following introduction episodes (Barrett and Husband 1990). We did not detect such a trend in Southern Italian and Sicilian populations, with the exception of few individuals that could represent the signature of old introductions from Balkans and Crete (Fig. 4). Indeed, both assignment tests based on STRUCTURE analysis and pattern in NJ tree (Figs 4 and 5) grouped the southern Italian and the Sicilian populations in independent lineages, related to Balkan populations, but not nested within. This evidence supports the hypothesis of autochthonous origin for southern Italian and Sicilian populations. Otherwise, this is congruent with a scenario of repeated introductions with a large number of founder genotypes from Balkan populations not sampled here. A comparative analysis of plastid DNA variation, maternally inherited and dispersed by seeds, could allow to verify migration trajectories and the location of source populations so providing a further independent line of evidence supporting or rejecting these contrasting hypotheses.

Apart for the potential contribution of ancient introductions, in more recent time, *P. hispanica* has been widely introduced across Europe and Asia as an ornamental tree within the original *P. orientalis* range (Besnard *et al.* 2002). Nevertheless, besides morphological differences (Tutin and Edmondson 1993), *P. orientalis* has a distinct microsatellite profile from introduced *P. hispanica* (Lang 2010; Johnson *et al.* 2016) that rule out the risk of incorrect assignment of our sampled populations. Further, estimations of putative introgression and of ongoing gene flow between introduced *P. hispanica* and natural *P. orientalis* stands revealed that, in contrast to what found in *P. racemosa* (Johnson *et al.* 2016), hybridization with introduced *P. hispanica* does not represent yet a threat to *P. orientalis.* Despite being wind-pollinated, *P. orientalis* pollen occurs mainly within 800 m from the source plant (Bricchi *et al.* 2000). The occurrence of *P. orientalis* populations in remote locations often very distant from *P. hispanica*, and the very low (if any) regeneration found in natural marginal populations, should protect *P. orientalis* from introgression and potential risk of genetic erosion by the introduced ornamental hybrid.

In conclusion, our study highlights that *P. orientalis* populations have high genetic differentiation and low gene flow, particularly at western edge, which is determined mainly by geographical isolation linked to its relict distribution. These results show that quantifying the population genetic variation of geographically disjunct species can yield insight into the mechanisms underlying their distributions, and help better understand their ability to colonize novel habitats in future changing environments.

## Data

An xlsx file of microsatellite genotype data set.

## Sources of Funding

This study was partially supported by a bilateral mobility grant CNR-Bulgarian Academy of Science.

## Contributions by the authors

S.S. and S.F. planned and designed the project; R.R. and D.C. conducted the experiments, F.S. ran the analyses; S.S., A.C. and S.F. wrote most of the text. All authors contributed in the preparation of the study and have commented on and approved the final manuscript.

## Conflict of Interest

None declared.

## Supplementary Material

Supplementary Table S1

Supplementary Table S2

Supplementary Table S3

Supplementary Table S4

Supplementary Figure S1

Supplementary Figure S2

Supporting Information

## Supporting Information

The following additional information is available in the online version of this article—

**Table S1.** A table with effective population sizes for *Platanus orientalis* populations.

**Table S2.** A table with the effective number of immigrants per generation, calculated for all sampled *Platanus orientalis* populations.

**Table S3.** A table with the microsatellite genotypes of putative hybrid samples detected in the examined *Platanus orientalis* populations.

**Table S4.** A table with the microsatellite genotypes of a *Platanus hispanica* commercial stock and of *P. hispanica* street trees collected in proximity of the Alento population.

**Figure S1.** A picture of the STRUCTURE output for *Platanus orientalis* populations including clonal individuals of Alento population.

**Figure S2.** A picture of the spatial distribution of genetic admixture considering the clusters from the software STRUCTURE.
