# Supplementary material for: The influence of a relict distribution on genetic structure and variation in the Mediterranean tree, Platanus orientalis
Source: AoB Plants. 2019 Jan 30;11(1):plz002. doi: 10.1093/aobpla/plz002 (PMC6381769; doi:10.1093/aobpla/plz002)
Supplement: Supplementary Table S1 [file plz002_suppl_supplementary_table_s1.pdf]

**Supplementary Table S1:** Effective population sizes including 95% confidence intervals.

| Populations | $N_e$[1] |
|---|---|
| ALE | 241.09 (203.6-399.5) |
| VEL | 198.60 (163.2-341.8) |
| CAL | 253.87 (215.7-407.4) |
| COS | Na |
| ALC | Na |
| ANA | 341.86 (314.8-497.1) |
| CAT | 336.15 (312.3-494.8) |
| MAN | 364.78 (312.5-512.7) |
| AUG | 512.23 (406.4-837.1) |
| GOR | 426.74 (357.9-689.4) |
| NES | 402.18 (331.7-671.3) |
| ACH | 261.54 (236.7-447.2) |
| VJA | 328.49 (268.6-484.6) |
| AOO | 467.04 (368.7-772.1) |
| OSU | 397.43 (342.8-767.9) |
| DRI | 356.82 (301.7-521.5) |
| VJB | Na |
| KRE | 354.27 (313.6-535.8) |
| TOP | 379.07 (314.8-667.9) |
| MIK | 458.61 (354.2-699.6) |
| DAR | 408.34 (336.8-712.3) |
| BES | 476.28 (352.3-798.1) |

[1]Effective population sizes were calculated using the formula $Ne = \text{theta}/4\mu$, where $\mu$ = mutation rate.
