# Supplementary material for: The influence of a relict distribution on genetic structure and variation in the Mediterranean tree, Platanus orientalis
Source: AoB Plants. 2019 Jan 30;11(1):plz002. doi: 10.1093/aobpla/plz002 (PMC6381769; doi:10.1093/aobpla/plz002)
Supplement: Supplementary Table S2 [file plz002_suppl_supplementary_table_s2.pdf]

Supplementary Table S2. The effective number of immigrants per generation, calculated for all sampled populations. The direction of gene flow is from row to column; NA= Not Available due to small population size. See Table 1 for population codes.

	ALE	VEL	CAL	COS	ALC	ANA	CAT	MAN	AUG	GOR	NES	ACH	VJA	AOO	OSU	DRI	VJB	KRE	TOP	MIK	DAR	BES
ALE		39.92	32.46	NA	NA	35.24	30.22	74.71	65.31	42.30	51.09	74.35	41.93	103.66	34.88	30.47	NA	22.22	23.27	15.77	36.22	36.39
VEL	16.57		30.24	NA	NA	27.77	27.96	27.16	38.74	27.59	48.90	16.96	28.87	34.34	33.23	24.35	NA	34.41	28.95	33.71	19.26	21.55
CAL	18.53	56.83		NA	NA	22.54	15.44	33.17	34.60	30.97	46.85	13.31	19.79	27.42	33.14	14.85	NA	29.10	22.18	47.98	34.51	25.13
COS	NA	NA	NA		NA	NA	NA	NA	NA	NA	NA	NA	NA	NA	NA	NA	NA	NA	NA	NA	NA	NA
ALC	NA	NA	NA	NA		NA	NA	NA	NA	NA	NA	NA	NA	NA	NA	NA	NA	NA	NA	NA	NA	NA
ANA	91.27	28.99	35.04	NA	NA		47.21	76.87	85.14	65.48	38.33	30.34	30.92	51.53	31.40	29.32	NA	66.04	26.62	56.41	21.13	18.47
CAT	19.37	39.71	35.52	NA	NA	22.54		59.19	24.56	47.21	21.87	18.83	33.41	22.87	33.54	21.44	NA	83.98	30.46	37.86	29.61	24.08
MAN	14.59	24.32	31.58	NA	NA	12.61	23.16		30.90	23.23	39.79	15.77	28.54	47.34	54.29	15.00	NA	30.42	36.65	29.67	34.67	18.98
AUG	32.86	39.62	45.00	NA	NA	41.83	23.66	28.46		37.34	42.69	27.43	33.48	24.52	28.59	16.84	NA	40.00	22.41	55.82	50.38	38.55
GOR	16.33	32.86	23.54	NA	NA	14.43	32.39	60.31	51.07		21.42	19.99	50.80	15.88	20.55	18.63	NA	30.04	36.56	32.66	34.48	14.13
NES	8.63	32.10	41.48	NA	NA	17.26	17.10	65.64	54.09	33.64		17.74	44.05	54.34	24.01	22.96	NA	64.22	40.71	42.54	36.33	17.79
ACH	28.76	27.10	33.32	NA	NA	30.34	20.46	45.91	68.26	38.44	70.10		36.19	51.08	42.90	30.90	NA	28.92	22.70	38.78	23.21	24.68
VJA	16.88	70.50	25.14	NA	NA	16.78	21.55	53.13	23.59	43.62	33.39	18.16		31.09	36.48	21.86	NA	28.04	42.15	54.93	37.34	24.87
AOO	14.98	38.84	30.83	NA	NA	15.19	15.77	141.37	88.10	15.38	64.60	19.43	42.45		27.22	16.66	NA	42.13	24.88	49.71	35.09	24.90
OSU	11.35	50.75	20.06	NA	NA	16.90	25.79	40.97	20.79	20.52	32.28	10.34	54.99	86.32		23.56	NA	48.13	22.36	42.48	33.25	26.21
DRI	25.73	16.13	34.40	NA	NA	29.22	21.91	35.22	46.43	21.11	44.35	22.96	21.41	59.67	20.80		NA	25.02	42.60	103.74	23.18	39.55
VJB	NA	NA	NA	NA	NA	NA	NA	NA	NA	NA	NA	NA	NA	NA	NA	NA		NA	NA	NA	NA	NA
KRE	10.17	20.93	64.30	NA	NA	32.45	33.34	63.95	41.66	33.29	56.22	22.34	45.10	30.01	23.77	16.82	NA		27.21	40.62	36.90	20.02
TOP	14.60	47.88	29.81	NA	NA	29.22	26.85	28.83	33.10	32.36	34.78	21.82	26.09	52.96	28.94	16.18	NA	28.15		45.82	35.73	32.43
MIK	12.41	36.94	37.62	NA	NA	17.35	22.91	40.30	50.74	14.73	60.00	24.61	24.31	56.58	29.49	20.23	NA	21.58	20.98		24.92	22.43
DAR	11.82	19.60	46.29	NA	NA	17.24	20.27	53.84	62.57	34.82	28.91	17.09	32.99	30.43	30.77	24.94	NA	28.90	37.28	26.48		40.52
BES	14.31	68.57	25.25	NA	NA	20.11	20.80	23.66	24.68	19.46	28.81	13.95	32.01	24.20	24.14	14.16	NA	25.42	34.63	83.03	74.23	
