# Supplementary material for: The influence of a relict distribution on genetic structure and variation in the Mediterranean tree, Platanus orientalis
Source: AoB Plants. 2019 Jan 30;11(1):plz002. doi: 10.1093/aobpla/plz002 (PMC6381769; doi:10.1093/aobpla/plz002)
Supplement: Supplementary Table S4 [file plz002_suppl_supplementary_table_s4.pdf]

Supplementary Table S4: Microsatellite genotypes of a *P. hispanica* commercial stock and of *P. hispanica* street trees collected in proximity of the Alento population. In bold the exclusive alleles not detected in the examined *P. orientalis* populations.

Source and location	plms29	plms68	plms71	plms109	plms113	plms130	plms176	11FAM	PI2A
Commercial stock	**218, 220**	**188, 190**	135, 135	123, 143	220, 220	208, 212	**265, 275**	230, 230	**358, 358**
Commercial stock	210, **230**	**190, 192**	135, 135	123, **161**	210, **230**	214, 214	275, **277**	230, 230	346, 348
Commercial stock	**218, 230**	**190, 192**	135, 135	123, **159**	**218, 230**	212, 214	**265**, **265**	232, 232	354, 354
Commercial stock	**218, 220**	182, **190**	135, 135	123, **161**	220, 220	208, 208	**265, 275**	204, 204	**356, 358**
Commercial stock	**218, 230**	**190, 192**	135, 135	123, **157**	**218, 230**	208, 208	275, **285**	230, 230	346, 348
Commercial stock	**218, 218**	**188, 190**	135, 135	123, **159**	**218, 218**	208, 212	**265,** 271	230, 230	**358, 358**
Commercial stock	**218, 218**	**188, 190**	135, 135	123, 123	212, **218**	208, 212	275, **275**	230, 230	352, **358**
Commercial stock	**218, 230**	**190, 190**	135, 135	145, **159**	220, **230**	208, 212	271, **275**	230, 230	350, 350
S-Italy, Alento street	208, **224**	**190, 190**	135, 135	123, 145	224, 226	208, 212	**265**, 269	226, 230	344, 354
S-Italy, Alento street	208, **224**	**190, 190**	135, 135	123, 145	224, 226	208, 212	**265**, 269	226, 230	344, 354
S-Italy Naples street	216, **224**	**190, 192**	135, 135			**204,** 208	279, 279	226, 230	354, 354
S-Italy Naples street	224, **224**	**192, 192**	135, 135	123, 143	220, 220	**204,** 210	**263,** 271	226, 230	350, 354
S-Italy Pagani street	208, **224**		135, 135	123, 143	208, 224	**204,** 208	**263, 267**	226, 230	344, 354
