# Supplementary material for: The influence of a relict distribution on genetic structure and variation in the Mediterranean tree, Platanus orientalis
Source: AoB Plants. 2019 Jan 30;11(1):plz002. doi: 10.1093/aobpla/plz002 (PMC6381769; doi:10.1093/aobpla/plz002)
Supplement: Supplementary Figure S1 [file plz002_suppl_supplementary_figure_s1.pdf]

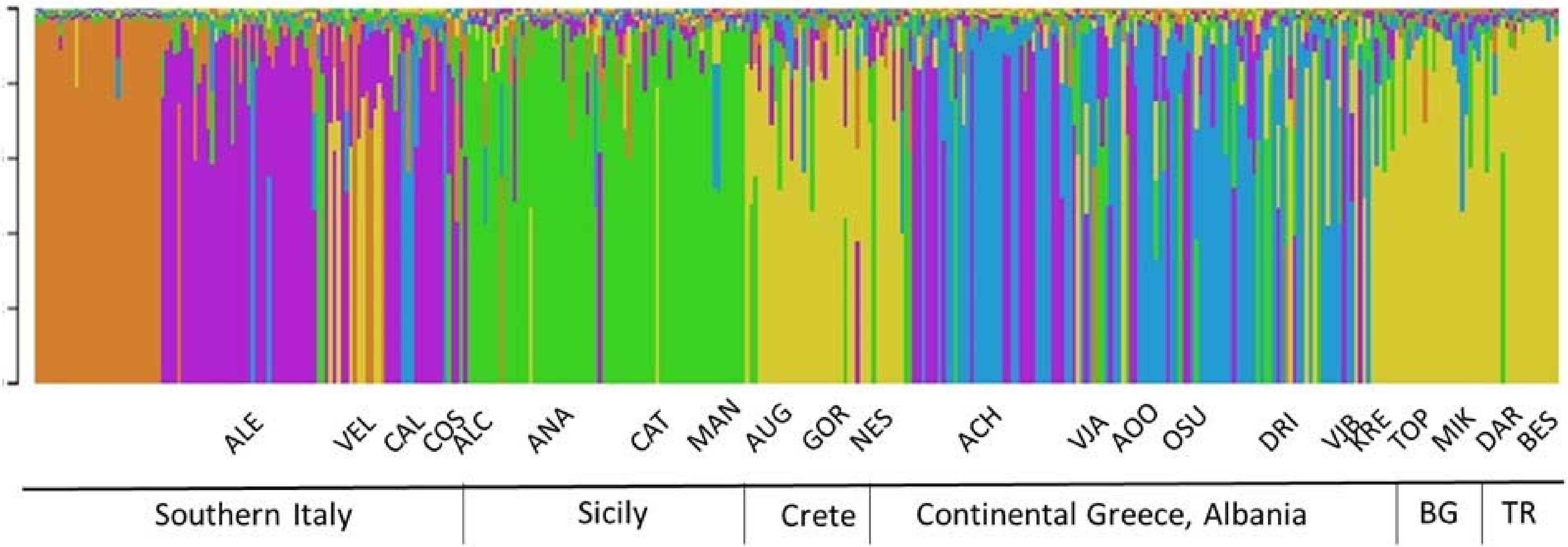

[Supplementary Figure S1]: Distruct plot for *Platanus orientalis* populations including clonal individuals of Alento population. Each cluster (K=5) is represented by a different color. See Table 1 for population codes.
