# Supplementary material for: The influence of a relict distribution on genetic structure and variation in the Mediterranean tree, Platanus orientalis
Source: AoB Plants. 2019 Jan 30;11(1):plz002. doi: 10.1093/aobpla/plz002 (PMC6381769; doi:10.1093/aobpla/plz002)
Supplement: Supplementary Figure S2 [file plz002_suppl_supplementary_figure_s2.pdf]

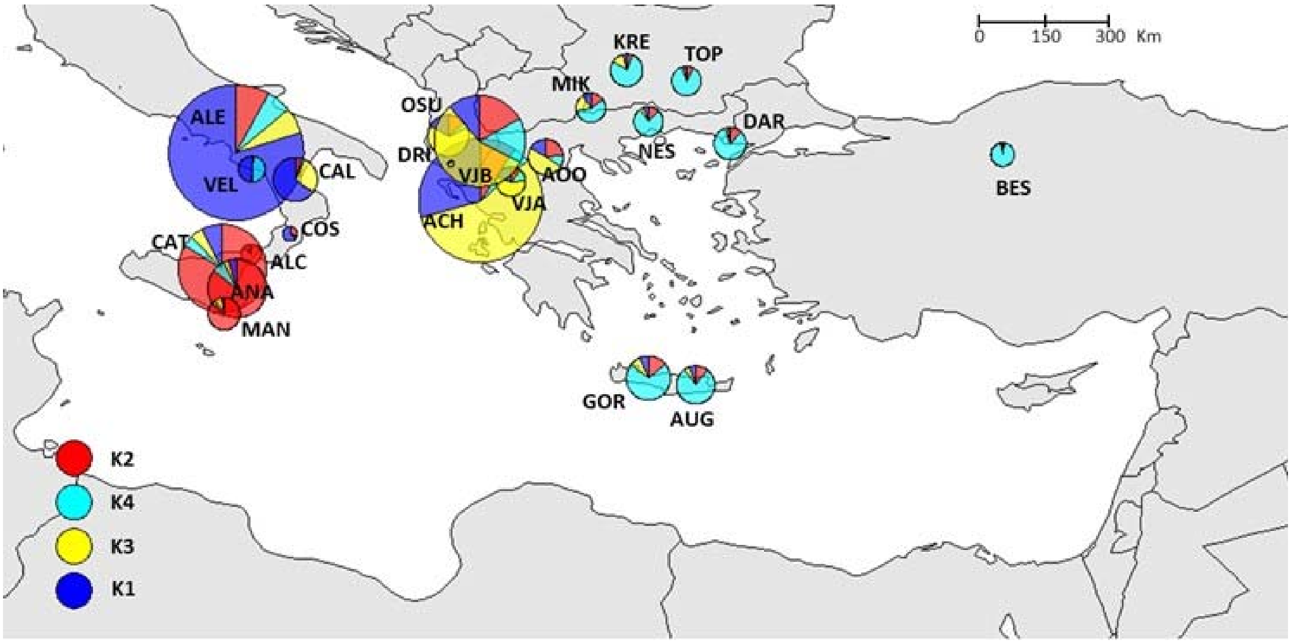

[Supplementary Figure S2]: Spatial distribution of genetic admixture considering the clusters from the software Structure. Each cluster (K= 4) is represented by a different color. See Table 1 for population codes.
